# [^18^F]FDG PET-MR characterization of aortitis in the *IL1rn*^*−/−*^ mouse model of giant-cell arteritis

**DOI:** 10.1186/s13550-023-01039-5

**Published:** 2023-11-29

**Authors:** Samuel Deshayes, Caroline Baugé, Pierre-Antoine Dupont, Christophe Simard, Hanan Rida, Hubert de Boysson, Alain Manrique, Achille Aouba

**Affiliations:** 1 Department of Internal Medicine and Clinical Immunology, Normandie University, UNICAEN, CHU de Caen Normandie – Université Basse Normandie, Avenue de la Côte de Nacre, 14000 CAEN, France; 2 Normandie University, UNICAEN, CHU de Caen Normandie, UR4650 PSIR Caen, France; 3 Department of Nuclear Medicine, Normandie University, UNICAEN, CHU de Caen Normandie, Caen, France

**Keywords:** *IL1rn*^*−/−*^ aortitis mouse model, Giant-cell arteritis, Aortitis, Interleukin-1, PET-MR

## Abstract

**Background:**

Metabolic imaging is routinely used to demonstrate aortitis in patients with giant-cell arteritis. We aimed to investigate the preclinical model of aortitis in BALB/c *IL1rn*^*−/−*^ mice using [^18^F]fluorodeoxyglucose ([^18^F]FDG) positron emission tomography–magnetic resonance (PET-MR), gamma counting and immunostaining. We used 15 first-generation specific and opportunistic pathogen-free (SOPF) 9-week-old *IL1rn*^*−/−*^ mice, 15 wild-type BALB/cAnN mice and 5 s-generation specific pathogen-free (SPF) 9-week-old *IL1rn*^*−/−*^. Aortic [^18^F]FDG uptake was assessed as the target-to-background ratio (TBR) using time-of-flight MR angiography as vascular landmarks.

**Results:**

[^18^F]FDG uptake measured by PET or gamma counting was similar in the first-generation SOPF *IL1rn*^*−/−*^ mice and the wild-type group (*p* > 0.05). However, the first-generation *IL1rn*^*−/−*^ mice exhibited more interleukin-1β (*p* = 0.021)- and interleukin-6 (*p* = 0.019)-positive cells within the abdominal aorta than the wild-type mice. In addition, the second-generation SPF group exhibited significantly higher TBR (*p* = 0.0068) than the wild-type mice on the descending thoracic aorta, unlike the first-generation SOPF *IL1rn*^*−/−*^ mice.

**Conclusions:**

In addition to the involvement of interleukin-1β and -6 in *IL1rn*^*−/−*^ mouse aortitis, this study seems to validate [^18^F]FDG PET-MR as a useful tool for noninvasive monitoring of aortitis in this preclinical model.

## Background

Giant-cell arteritis (GCA) is the most frequent systemic vasculitis among people over 50 years of age [1]. This disease is estimated to affect more than 3 million people by 2050 [2]. Aortitis is diagnosed in 40–80% of GCA patients and is associated with a poor prognosis, mainly related to aneurysms and aortic dissection [3–7]. Corticosteroids fail to significantly inhibit the Th1 pathway and vascular remodelling factors [8, 9]. They therefore appear most often unable to promptly control aortic inflammation and the onset of subsequent complications, as shown by repetitive [^18^F]fluorodeoxyglucose ([^18^F]FDG) positron emission tomography (PET) [10, 11]. GCA patients under anti-interleukin (IL)-6 therapy, which demonstrated a considerable steroid-sparing effect in GCA, may also exhibit persistent [^18^F]FDG aortic uptake on PET imaging. However, these features can correspond either to persistent active inflammation or to vascular remodelling [12].

The pathophysiology of GCA remains poorly understood and mainly relies on studies using blood and temporal artery samples obtained from GCA patients [13]. Pathophysiological data on aortic involvement are very scarce and prevent detailed knowledge of the efficacy of current treatments and the development of new targeted therapies. BALB/c *IL1rn*^*−/−*^ mice, mutated in the gene encoding the natural IL-1 receptor antagonist, represent one of the few available preclinical models of aortitis [13]. Indeed, BALB/c *IL1rn*^*−/−*^ mice develop transmural aortitis with a neoangiogenic process, from aortic root to popliteal arteries, mimicking human GCA, affecting half of the mice that are 8 weeks old, 78% of mice at a median age of 147 days in the BALB/c background, and 100% of mice at 200 days in the Sf3 background, and associated with a risk of death resulting from an infarction or ruptured aneurysms [14–17]. Preclinical metabolic imaging using [^18^F]FDG PET-CT allows serial in vivo imaging of inflammation and/or therapeutic interventions and may help understand temporal fluctuations of the disease. However, quantitative or semiquantitative analysis of aortic glucose uptake warrants anatomical landmarks and requires further CT contrast injection to visualize vascular structures. Hybrid PET-MR is a novel technique that allows non-contrast MR angiography to visualize vascular structures without increasing the injected volumes in small animals. Metabolic imaging using PET-MR has never been proposed in BALB/c *IL1rn*^*−/−*^ mice to improve the knowledge on the time course of vascular inflammation and thus the therapeutic perspectives in GCA. Therefore, in this work, we aimed to characterize, on the one hand, the pattern of proinflammatory cytokines involved in immunostaining and, on the other hand, the profile of aortic metabolic imaging using [^18^F]FDG PET-MR in 9-week-old BALB/c *IL1rn*^*−/−*^ mice.

## Methods

### Mice

BALB/c *IL1rn*^*−/−*^ (CAnN.129P2(MF1)-Il1rn^tm1Nick^/NickH) frozen sperm was obtained from the M.R.C. Harwell Institute, which distributes this strain on behalf of the European Mouse Mutant Archive (EMMA: www.infrafrontier.eu). The repository number is EM:02497. The CAnN.129P2(MF1)-Il1rn^tm1Nick^/NickH mice were originally produced at the University of Sheffield [15–18]. Specific and opportunistic pathogen-free (SOPF) mice were rederived at Janvier Labs (Le Genest St. Isle, France) by in vitro fertilization using BALB/c *IL1rn*^*−/−*^ frozen sperm and oocytes from heterozygous BALB/cAnN *IL1n*^*+/−*^ female mice from an initial revitalization. A control group was constituted of 15 wild-type specific pathogen-free (SPF) BALB/cAnN mice (Janvier Labs). As previous studies did not find any difference between males and females regarding the incidence of aortitis in this model [14, 17], we chose to use both in our experiments.

To better refine our data, the second group of 5 BALB/c *IL1rn*^*−/−*^ mice, resulting from the mating of the first-generation mice but in an SPF environment in the Centre Universitaire de Ressources Biologiques (CURB, Caen, France), also underwent PET-MR at 9 weeks. These mice were part of a longitudinal study.

All animal procedures were performed according to the European directive 2010/63/EU on protecting animals used for scientific purposes and specific French laws (decree n°2013–118) and were approved by the regional animal ethics committee (Comité d'Éthique NOrmand en Matière d'EXpérimentation Animale, CENOMEXA 054, n°23,523). Mice were housed in an SPF environment in a temperature-controlled room with ad libitum access to standard mouse chow and water. The study is reported in accordance with ARRIVE guidelines.

Immediately after PET-MR, mice were euthanized by decapitation while under deep anaesthesia and unresponsive to all stimuli (5% isoflurane gas in an N_2_O/O_2_ mixture (2:1)). All precautions were taken to minimize suffering.

### PET-MR

Acquisitions were performed using a hybrid PET-MR 7 T system dedicated to small animals (BioSpec 70/18, Bruker, Ettlingen, Germany). For PET imaging, a static whole-body PET scan (15 min) was obtained. The energy windows were set to 357–664 keV, and the coincidence windows were set to 5 ns. The axial scan length was 117 mm. The image data were corrected for dead time, radioactive decay, attenuation, scatter, and randoms. Attenuation correction was based on segmenting tissue, air, and animal cradles using whole-body 3-dimensional FISP MR acquisition [19]. The sinograms were reconstructed using a 3-dimensional OSEM algorithm (12 iterations, 16 subsets) into a 128 × 128 matrix (slice thickness: 0.75 mm). The MR protocol consisted of a localizer, T2 TurboRARE (TE: 24 ms, TR: 1720 ms, 150  × 150 matrix, FOV: 30 × 30 mm, voxel size: 0.2 × 0.2 × 0.5, RARE factor: 5, averages: 8, slice thickness: 0.5 mm, slices: 30), 2D TOF FLASH MR angiography (TE: 1.657 ms, TR: 12 ms, 125  × 125 matrix, bandwidth: 700 Hz/pixel, flip angle: 80°, averages: 2, slice thickness: 0.6 mm, slices: 60) and whole-body FISP (TE: 2.6 ms, TR: 5.5 ms, flip angle: 10°, image size: 80  × 80, averages: 3, slice thickness: 0.5 mm, slices: 60) sequences. The 2D TOF imaging was gated to the ECG to avoid cardiac motion artefacts, and T2 TurboRARE was gated to the respiratory signal. All 2D images were acquired in axial view. MR images were used as landmarks to quantify [^18^F]FDG uptake in vessels of interest. However, no qualitative or quantitative data regarding both the morphology and dimension of the aorta could be inferred from these images, because of the size of the vessels and the limited spatial resolution of MR angiography.

To decrease myocardial [^18^F]FDG uptake, 9-week-old conscious mice were fed a ketogenic diet for two days and then fasted 18 h before the PET-MR scan, and [^18^F]FDG was intraperitoneally injected (25.9 ± 1.3 MBq, Curium, Glisy, France) [20–22]. Fifty minutes later, the mice were anaesthetized with 5% isoflurane gas and maintained with isoflurane 2% gas in an N_2_O/O_2_ mixture (2:1) during the PET-MR procedure. MR sequences were obtained first, and PET images were acquired 83 ± 1 min after the [^18^F]FDG injection.

PET-MR analysis was performed using 3D Slicer 4.11 (http://www.slicer.org/) using the PET-IndiC extension [23–26]. A rigid transformation (6 degrees of freedom) was applied to align the reconstructed PET to MR images. Volumes of interest (VOIs) were manually drawn with the “Level Tracing Effect” tool in the three axes, encompassing the entire available portion of the ascending thoracic aorta, descending thoracic aorta, abdominal aorta, and inferior vena cava as identified on 2D TOF angiography. To include the vessel wall, the VOIs of the aorta were dilated by the neighbourhood method (8 neighbours). Aortic uptake within each VOI was expressed as the target-to-background ratio (TBR), defined as the ratio of the maximum uptake in the aorta (in Bq/mL) within each VOI of the aorta to the maximum uptake of the residual vascular activity measured in the inferior vena cava.

### Ex vivo radioactivity measurement

The aorta was removed, and a piece at the thoracoabdominal junction was taken, weighed, and gamma counted with a designated [^18^F] protocol for 60 s (Wizard 2470, PerkinElmer, Boston, MA, USA) 126 ± 2 min after the [^18^F]FDG injection. The results were expressed as counts per minute (CPM) per administered dose and milligram of tissue (CPM.MBq^−1^.mg^−1^). As the 5 SPF mice were part of a longitudinal study, these mice have not been euthanized at 9 weeks old and therefore no ex vivo radioactivity measurement was available.

### Phosphor imaging autoradiography

The remaining aorta was embedded in an optimal cutting embedding medium (OCT, Thermo Scientific, Waltham, USA) and stored at -80 °C. Thoracic and abdominal aortas were then longitudinally sectioned (10 µm), mounted on glass slides, and placed on a phosphor imaging plate (BAS-IP Phosphorimaging plate, GE Healthcare Life Sciences, Pittsburgh, PA, USA) 167 ± 4 min after [^18^F]FDG injection and then imaged with an Amersham Typhoon 5 (GE Healthcare Life Sciences) after overnight exposure and with a pixel resolution of 10 µm. No phosphor imaging autoradiography was performed for the 5 SPF mice, because these mice have not been euthanized at 9 weeks old.

### Histological staining of aortic samples

Routine staining was performed using haematoxylin, eosin, and saffron (HES). Immunostaining was performed on a BenchMark XT (Ventana Medical Systems Inc., Tucson, AZ, USA) with the following rabbit antibodies directed against immune cells or endothelial cells and according to the manufacturer’s protocols: anti-CD4 (1:50, Cell Signalling, catalogue number: #25229), anti-CD8α (1:200, Cell Signalling, catalogue number: #98941), anti-CD31 (1:100, Cell Signalling, catalogue number: #77699), anti-CD11c (1:100, Cell Signalling, catalogue number: #97585), anti-CD68 (1:600, Cell Signalling, catalogue number: #97778), anti-IL-17A (1:100, Thermo Fisher Scientific, catalogue number: #PA5-79470), anti-IL-6 (1:100, Thermo Fisher Scientific, catalogue number: #BS-0782R), anti-interferon-γ (1:100, Abcam, catalogue number: #ab216642), anti-CD163 (1:200, Abcam, catalogue number: #ab182422), anti-IL-1β (1:150, Abcam, catalogue number: #ab205924), anti-IL-1α (1:100, Abcam, catalogue number: #ab7632), anti-CD20 (1:50, Abcam, catalogue number: #ab64088). Bound antibodies were detected using avidin–biotin-peroxidase complex (ChromoMap DAB and OmniMap anti-Rabbit HRP, Roche Diagnostics, Meylan, France). Images were acquired using an Olympus VS120 slide scanner (Olympus, Tokyo, Japan) at 20 × magnification. Tissue segmentation was manually performed using the QuPath software package (v0.2.3) [27]. For HES and immunostaining, data were expressed as the mean number of cells per mm^2^ or as the mean number of positive cells per thousand using the “Cell detection” or the “Positive cell detection” option, respectively (optical density sum, Score compartment: Eosin OD max or DAB OD max with single threshold). Histological staining was not available for the 5 SPF mice, as these mice were part of a longitudinal study.

### Statistical analyses

Quantitative data are expressed as the mean ± standard error of the mean and were analysed using the Mann–Whitney test. Associations were considered significant if the p value was < 0.05. Statistical analyses were performed using GraphPad Prism 7 (GraphPad Software Inc., San Diego, CA, USA). The association between TBR and mouse groups (wild-type mice, first- or second-generation *IL1rn*^*−/−*^ mice) or aortic segments (abdominal aorta, ascending or descending thoracic aorta) or aortic segments by mouse group interaction was analysed in a multiple linear regression by the least squares method conducted by JMP 11 (SAS Institute, Cary, NC).

## Results

The first-generation SOPF *IL1rn*^*−/−*^ and the wild-type mice were each composed of 8 females and 7 males who were 9 weeks old. The mean weight was 20.2 ± 0.7 g in the first-generation *IL1rn*^*−/−*^ group and 19.2 ± 0.6 g in the wild-type group (*p* = 0.24).

### PET-MR

**Fig. 1:**
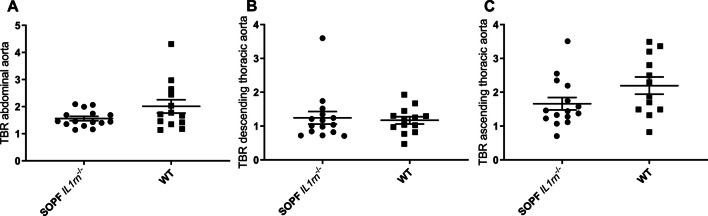
Results of [^18^F]FDG PET quantification in first-generation specific and opportunistic pathogen-free 9-week-old BALB/c *IL1rn*^*−/−*^ (*n* = 15) or wild-type mice (*n* = 13 or *n* = 12 for the ascending thoracic aorta). **A** Abdominal aorta, *p* = 0.26. **B** Descending thoracic aorta, *p* = 0.66. **C** Ascending thoracic aorta, *p* = 0.09

Due to technical issues, PET-MR analysis was not feasible in 2 mice and feasible only in the descending thoracic and abdominal aorta in one (all in the wild-type group). The results are depicted in Fig. 1. No significant differences were found between males and females, within the overall cohort or each group (*p* > 0.05).

**Fig. 2:**
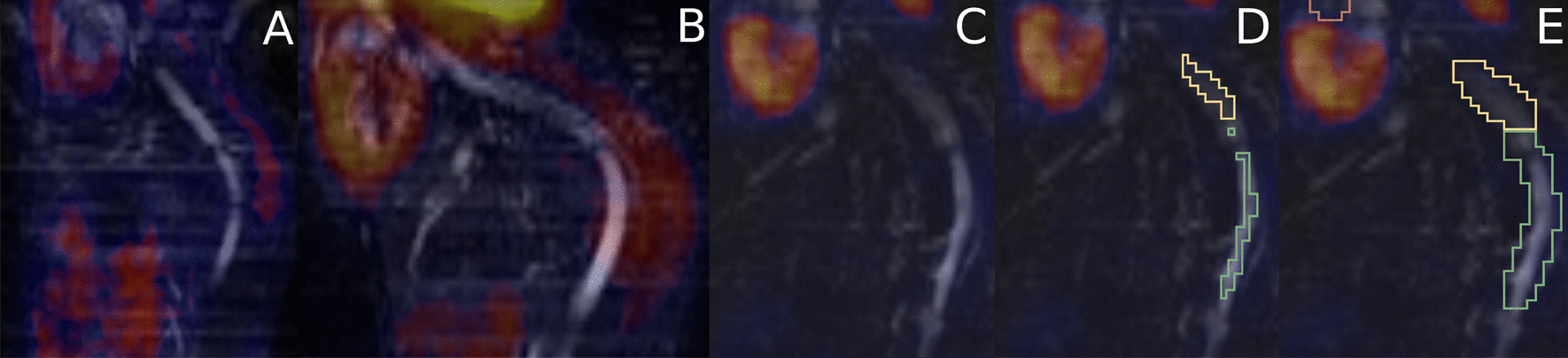
Representative images of [^18^F]FDG PET-MR (sagittal slices of 2D TOF FLASH MR angiography sequences through the descending thoracic and abdominal aorta and the heart) showing a normal physiological [^18^F]FDG uptake in the heart and the paravertebral brown adipose tissue, and an increased [^18^F]FDG uptake in the descending thoracic aorta in the second-generation BALB/c *IL1rn*^*−/−*^ mouse. **A** First-generation BALB/c *IL1rn*^*−/−*^ mouse. **B** Second-generation BALB/c *IL1rn*^*−/−*^ mouse. **C** Wild-type mouse. **D** Volumes of interest manually drawn (yellow: descending thoracic aorta; green: abdominal aorta). **E** Volumes of interest manually drawn after dilation by the neighbourhood method (yellow: descending thoracic aorta; green: abdominal aorta; pink: ascending thoracic aorta)

**Fig. 3:**
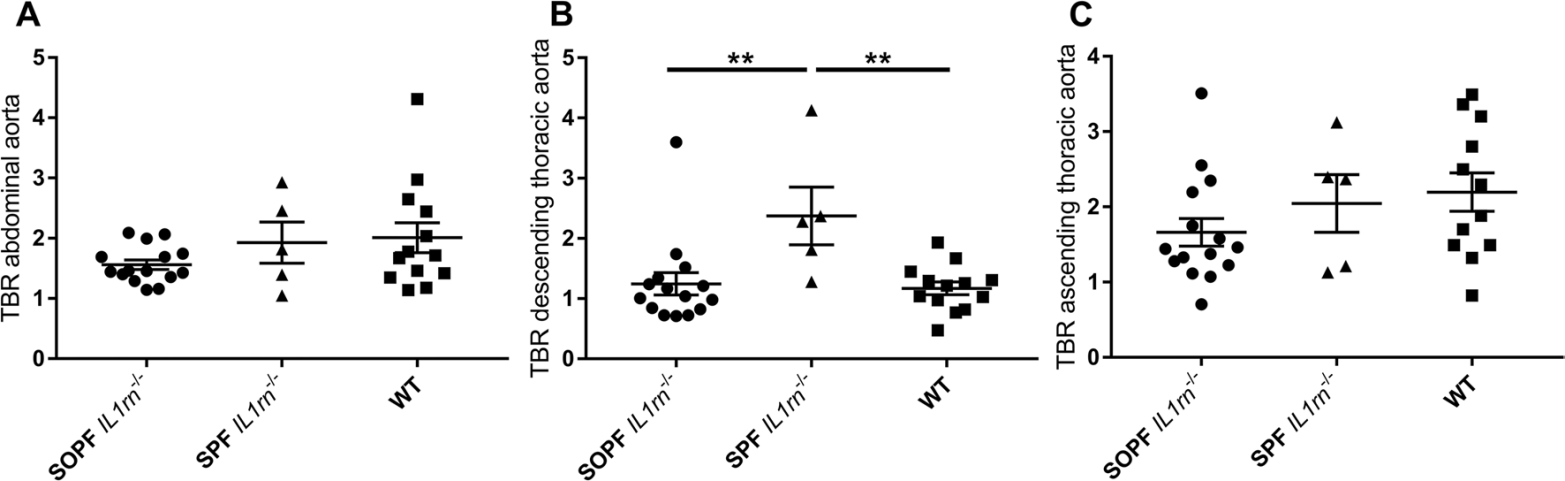
Results of [^18^F]FDG PET quantification in second-generation specific pathogen-free 9-week-old BALB/c *IL1rn*^*−/−*^ (*n* = 5) or wild-type mice (*n* = 13 or *n* = 12 for the ascending thoracic aorta). **A** Abdominal aorta, SPF *versus* WT, *p* = 1; SPF *versus* SOPF, *p* = 0.50. **B** Descending thoracic aorta, SPF *versus* WT, *p* = 0.0068; SPF *versus* SOPF, *p* = 0.0055. **C** Ascending thoracic aorta, SPF *versus* WT, *p* = 0.65; SPF *versus* SOPF, *p* = 0.50

However, the same imaging protocol was applied to 5 mice (19.6 ± 0.7 g) from the second generation after rederivation in an SPF environment. Compared to the first-generation SOPF *IL1rn*^*−/−*^ or to wild-type mice, the TBR was significantly higher in the descending thoracic aorta of the second-generation SPF *IL1rn*^*−/−*^ mice (*p* = 0.0055 and 0.0068, respectively, Figs. 2 and 3).

In the multiple linear regression analysis including all mice, the TBR was significantly associated with mouse groups (*p*  = 0.011) but not with the aortic segment (*p* = 0.18) or the interaction term (*p* = 0.082).

### Ex vivo radioactivity measurement and phosphor imaging autoradiography

No significant differences were found regarding the radioactivity measured by gamma counter (791.7 ± 167.4 in the first-generation *IL1rn*^*−/−*^ group vs. 1138.8 ± 225.1 CPM.MBq^−1^.mg^−1^ in the wild-type group, *p* = 0.29). Autoradiography showed aortic uptake of [^18^F]FDG in both groups.

### Histological staining

Regarding the immunostainings, there were significantly more positive cells marked by the anti-IL-1β and anti-IL-6 antibodies in the first-generation *IL1rn*^*−/−*^ group than in the wild-type group on abdominal aorta samples (*p* = 0.021 and 0.019, respectively, Table 1).

**Table 1:** Results of histological stainings of mice aorta

	*IL1rn*^*−/−*^ (*n* = 15)	Wild-type (*n* = 15)	*p* Value
*Thoracic aorta*			
CD4	5.16 ± 1.95	6.85 ± 1.69	0.38
CD8	6.08 ± 1.47	13.07 ± 6.09	0.63
CD11c	13.31 ± 2.64	9.29 ± 1.79	0.19
CD20	11.83 ± 3.33	9.53 ± 3.42	0.33
CD31	519.51 ± 18.52 (*n* = 14)	525.31 ± 21.68	0.92
CD68	10.83 ± 3.10	9.47 ± 1.96	0.97
CD163	10.43 ± 1.56	7.83 ± 2.27	0.07
Interferon-γ	100.71 ± 19.31	71.10 ± 14.40 (*n* = 14)	0.21
Interleukin-1α	39.82 ± 9.48	54.11 ± 15.81	0.57
Interleukin-1β	38.51 ± 9.96	41.22 ± 14.34	0.35
Interleukin-6	25.54 ± 10.00	21.45 ± 7.01 (*n* = 14)	0.95
Interleukin-17A	361.53 ± 24.98	347.04 ± 20.85	0.75
Haematoxylin–eosin-saffron stain^a^	8348.63 ± 295.7	8507.03 ± 274.2	0.55
*Abdominal aorta*			
CD4	1.50 ± 0.45 (*n* = 14)	3.96 ± 1.11	0.11
CD8	4.38 ± 1.50	2.69 ± 0.41	1
CD11c	13.19 ± 3.68	8.95 ± 2.18 (*n* = 14)	0.36
CD20	31.15 ± 10.14	16.61 ± 7.22 (*n* = 14)	0.41
CD31	597.56 ± 21.93 (*n* = 14)	584.45 ± 13.80	0.92
CD68	8.02 ± 2.01	9.83 ± 1.97	0.52
CD163	3.93 ± 1.32	4.41 ± 0.79	0.13
Interferon-γ	74.27 ± 11.69	55.84 ± 12.23	0.17
Interleukin-1α	48.53 ± 6.12	31.34 ± 6.22	0.056
Interleukin-1β	46.26 ± 16.52	11.88 ± 2.28	**0.021**
Interleukin-6	26.45 ± 5.17	13.44 ± 2.85	**0.019**
Interleukin-17A	363.19 ± 26.73	340.98 ± 12.67	0.88
Haematoxylin–eosin-saffron stain^a^	7964.49 ± 141.6	8175.01 ± 169.6	0.57
*Whole aorta*			
CD4	3.33 ± 0.95	5.32 ± 1.02	0.18
CD8	4.96 ± 0.92	10.31 ± 5.01	0.81
CD11c	12.51 ± 2.08	9.75 ± 1.68	0.42
CD20	19.05 ± 4.45	12.58 ± 4.52	0.17
CD31	537.08 ± 17.27	550.10 ± 17.74	0.39
CD68	10.08 ± 2.11	10.05 ± 1.70	0.66
CD163	7.65 ± 1.01	5.89 ± 1.08	0.14
Interferon-γ	85.39 ± 10.91	59.43 ± 8.99	0.098
Interleukin-1α	42.64 ± 8.05	51.19 ± 12.95	0.94
Interleukin-1β	31.19 ± 5.88	33.01 ± 11.75	0.31
Interleukin-6	25.36 ± 5.40	17.10 ± 4.16	0.25
Interleukin-17A	360.30 ± 21.28	345.98 ± 15.16	0.54
Haematoxylin–eosin-saffron stain^a^	8127.59 ± 161.7	8202.92 ± 185.1	0.54

Unless indicated, values are displayed as the mean number of positive cells per thousand (± SEM). Significant values (i.e. *p* value < 0.05) are displayed in bold

^a^Expressed as the mean number of cells per mm^2^ (± SEM)

## Discussion

To our knowledge, this is the first study assessing in vivo aortic inflammation using [^18^F]FDG PET-MR in the *IL1rn*^*−/−*^ mouse model of aortitis at 9 weeks old, combined with ex vivo gamma well counting and aortic wall cytokine expression. First, considering the first-generation mice, we did not find any significant differences regarding aortic [^18^F]FDG uptake measured in vivo by PET or ex vivo by gamma counter in 15 SOPF 9-week-old *IL1rn*^*−/−*^ mice when compared to wild type. However, we found a significantly higher number of IL-1β- and IL-6-positive cells in the *IL1rn*^*−/−*^ group within the abdominal aortic wall. This study, therefore, corroborates the preferential involvement of these two cytokines in the pathophysiology of this model, as previously observed in this mouse model and in GCA based on fundamental and targeted cytokine therapeutic data [15, 28–32]. Second, considering second-generation mice, the TBR measured on PET-MR was significantly higher in the descending thoracic aorta of five 9-week-old *IL1rn*^*−/−*^ mice born and housed in an SPF environment than in the wild-type group, suggesting a role of the microbiota, probably that in the gut [33], in the induction of inflammatory aortitis in this model.

Several data are in favour of the involvement of IL-1 in the pathophysiology of GCA, including in vitro data, mouse models, and studies on GCA patients, including the efficacy of anti-IL-1 treatment based on case reports [29, 30]. The *IL1rn*^*−/−*^ mouse model of aortitis has been well described by different teams and on different genetic backgrounds [14–17, 28, 34, 35], and spontaneous transmural inflammation at the aortic root has been described in up to 15% of wild-type BALB/c mice [36]. In this study, we failed to detect any significant differences between first-generation SOPF 9-week-old *IL1rn*^*−/−*^ and wild-type mice at 9 weeks old regarding the aortic [^18^F]FDG uptake measured in vivo by PET-MR or ex vivo by a gamma counter. In our opinion, the absence of high aortic [^18^F]FDG uptake in this mouse model may be explained by the following two hypotheses. First, the evaluation at 9 weeks old was probably too early for the onset of clear histological and imaging translation of aortitis. This assumption is supported by the higher number of IL-1β- and IL-6-positive cells by immunostaining within the abdominal aortic wall in the first-generation *IL1rn*^*−/−*^ group, suggesting that the early steps of aortitis development were set up as no increased cell infiltration into the vascular wall was found. However, because of the lack of histological data in the second-generation mice, this assumption cannot be tested. Moreover, some studies have described the onset of aortitis as early as four weeks in the BALB/c background [14], and the half-life of aortitis onset has been estimated at 52 days in the Sf3 background [17]. Second, and as a corollary to the first hypothesis, we assume that this increased onset delay in our study is probably due to other independent procedural and/or environmental factors. In particular, the modification of the gut microbiota induced by the rederivation of the line, as we used the first generation of rederived homozygous SOPF *IL1rn*^*−/−*^ mice [37–40], appears to be the most likely candidate to explain our findings in the second-generation mice, according to our experimental conditions. Indeed, microbiota, including gut microbiota, dramatically impacts systemic and local immunities and dysbiosis has been implicated in several inflammatory diseases [41, 42], including in primary vasculitis (Kawasaki disease, ANCA-associated vasculitis, Behçet’s disease)[43–46]. In addition, a specific blood microbiome profile was found in GCA patients, which is considered a reflection of other microbiota [47]. Recent studies have shown specific microbiomes in temporal arteries and in thoracic aorta aneurysms of GCA patients [48, 49]. To our knowledge, the effect of gut microbiota on the development or on specific characteristics of aortitis has not been studied, although Nicklin et al. [15] specified that some mouse colonies develop aortitis earlier and that mice raised under quarantine or specific pathogen-free conditions also develop aortitis. Moreover, Rogier et al. [33] previously showed in the *IL1rn*^*−/−*^ model that the onset of spontaneous arthritis was dependent on the gut microbiota, with the rheumatic symptoms being decreased under germ-free conditions and further suppressed under tobramycin therapy. In line with these results, in our second-generation 9-week-old *IL1rn*^*−/−*^ mice born and raised under SPF conditions, the TBR of [^18^F]FDG was significantly higher in the descending thoracic aorta than in the wild-type group. However, we did not collect mouse faeces before euthanasia to assess microbiota differences between these two generations of mice.

The lack of this last procedure is one of the limitations of our study to fully assess all the fields of the *IL1rn*^*−/−*^ mouse model. These results are based on a small number of mice, especially regarding the SPF group, and need to be replicated with longitudinal data. In addition, we did not have the possibility to obtain histological data or ex vivo radioactivity measurement in the 5 SPF mice, because these mice were part of a longitudinal study, which prevented us from making correlations between increased [^18^F]FDG uptake, found in second-generation mice, with histological data, only available from the first-generation group. If these data do not allow a conclusion, they evoke a hypothesis that allows the interpretation of the rest of the experiment. In addition, the experimental plan of this model could be extended by assessing the efficacy of treating aortitis with various candidate drugs. Indeed, the various combinations of anti-IL-1, anti-IL-6, anti-IL-17, anti-IL-12/23 or anti-Janus kinase drugs, different doses of corticosteroids, and/or conventional immunosuppressants could be proposed as preventive and/or curative treatments in this model to preselect the best candidates for therapeutic trials in human GCA. Future investigations should focus on these perspectives after the present preliminary study demonstrates that this model is robust and suitable.

## Conclusions

This first study, including [^18^F]FDG PET-MR in the *IL1rn*^*−/−*^ mouse model of aortitis at 9 weeks old, demonstrates the fit of this *IL1rn*^*−/−*^ mouse model with GCA for the key pro-inflammatory and pathogenic cytokines. We demonstrated the feasibility of in vivo assessment of aortic inflammation using [^18^F]FDG in conjunction with MR angiography without contrast injection using hybrid PET-MR. Concomitantly, although based on a small number of mice, we showed significantly increased [^18^F]FDG uptake in PET-MR on the descending thoracic aorta of second-generation SPF *IL1rn*^*−/−*^ mice but not in first-generation SOPF *IL1rn*^*−/−*^ mice compared to wild-type mice. These findings may suggest the possible effect of the gut microbiota on the pathophysiology of aortitis in this model, as previously shown for spontaneous arthritis [33]. These data show that PET-MR is an interesting noninvasive method to monitor aortitis in this model and paves the way for the preclinical assessment of new drugs in this model that would help in the development of new treatment strategies in human GCA.

## Data Availability

All raw data are available from the Open Science Framework database (https://osf.io/yvf2j).

## Abbreviations

CPM: Counts per minute
FDG: Fluorodeoxyglucose
HES: Haematoxylin, eosin, and saffron
IL: Interleukin
GCA: Giant-cell arteritis
PET: Positron emission tomography
SPF: Specific pathogen-free
SOPF: Specific and opportunistic pathogen-free
TBR: Target-to-background ratio
VOI: Volumes of interest
